# Lysophospholipid Signaling Molecules in Fundus Neovascular Diseases

**DOI:** 10.3390/ijms272110025

**Published:** 2026-11-09

**Authors:** Xinbei Zhou, Shaolang Chen, Jiajian Liang, Junchao Chen, Hongan Hu, Wai Kit Chu, Qingping Liu, Changzhen Fu

**Affiliations:** 1 Joint Shantou International Eye Center of Shantou University and The Chinese University of Hong Kong, Shantou 515041, China; 24xbzhou@stu.edu.cn (X.Z.); csl@jsiec.org (S.C.); ljj@jsiec.org (J.L.); 25jcchen@stu.edu.cn (J.C.); 25hahu@stu.edu.cn (H.H.); waikit@cuhk.edu.hk (W.K.C.); 2 Fifth Clinical Institute of Shantou University Medical College, Shantou 515041, China; 3 Department of Ophthalmology & Visual Sciences, The Chinese University of Hong Kong, Hong Kong SAR 999077, China

**Keywords:** fundus neovascular diseases, lysophospholipid signaling molecules, lysophosphatidic acid, lysophosphatidylcholine, sphingosine-1-phosphate

## Abstract

Fundus neovascular diseases (FNDs) are vision-threatening ocular disorders featuring pathological neovascularization and pose a major global threat to vision health. Their pathogenesis is heterogeneous, involving multiple factors and pathways. Current clinical pharmacotherapies only target a limited range of molecular pathways via narrow mechanisms, leading to frequent suboptimal responses and disease recurrence, which restricts further therapeutic improvement for FNDs. Recent studies demonstrate that lysophospholipid signaling molecules selectively activate multiple cell-surface receptors, including G protein-coupled receptors, triggering multilayered downstream signaling cascades that govern neovascular initiation and progression. Aberrant activation of this endogenous signaling axis may underpin the limited efficacy of existing anti-angiogenic therapies, and its roles in pathological neovascularization have attracted intensive research attention. This review addresses three representative lysophospholipid mediators: lysophosphatidic acid, lysophosphatidylcholine, and sphingosine-1-phosphate. We systematically summarize their metabolic routes, receptor binding profiles, downstream signaling networks, and pro-angiogenic mechanisms. We also discuss the current research status and translational potential of targeting lysophospholipid axes as innovative therapies, aiming to provide theoretical and translational evidence for developing precise pathway-directed interventions.

## 1. Introduction

Fundus neovascular diseases (FNDs), a group of blinding ocular disorders characterized by pathological neovascularization, such as neovascular age-related macular degeneration (nAMD), proliferative diabetic retinopathy (PDR), and retinal vein occlusion (RVO), are a leading cause of irreversible vision loss worldwide [1,2,3,4]. Vascular endothelial growth factors (*VEGFs*), angiopoietins (*Angs*), dysregulated lipid metabolism, oxidative stress, and chronic inflammation, among other heterogeneous pathogenic factors, are recognized as key drivers of FNDs [4,5,6,7]. Currently, the therapeutic targets of first-line clinical interventions are primarily *VEGFs* and *Ang-2*. Although these approaches have demonstrated substantial in suppressing neovascular progression and maintaining central vision, their effectiveness remains constrained by the multifactorial and multipathway nature of FND pathogenesis. Approximately 25% of patients exhibit primary non-response or suboptimal response to initial treatment, and recurrence rates during long-term follow-up remain as high as 73.9%, highlighting the fundamental limitations of current therapeutic strategies in addressing the heterogeneous etiologies underlying FNDs [8,9].

**Figure 1:**
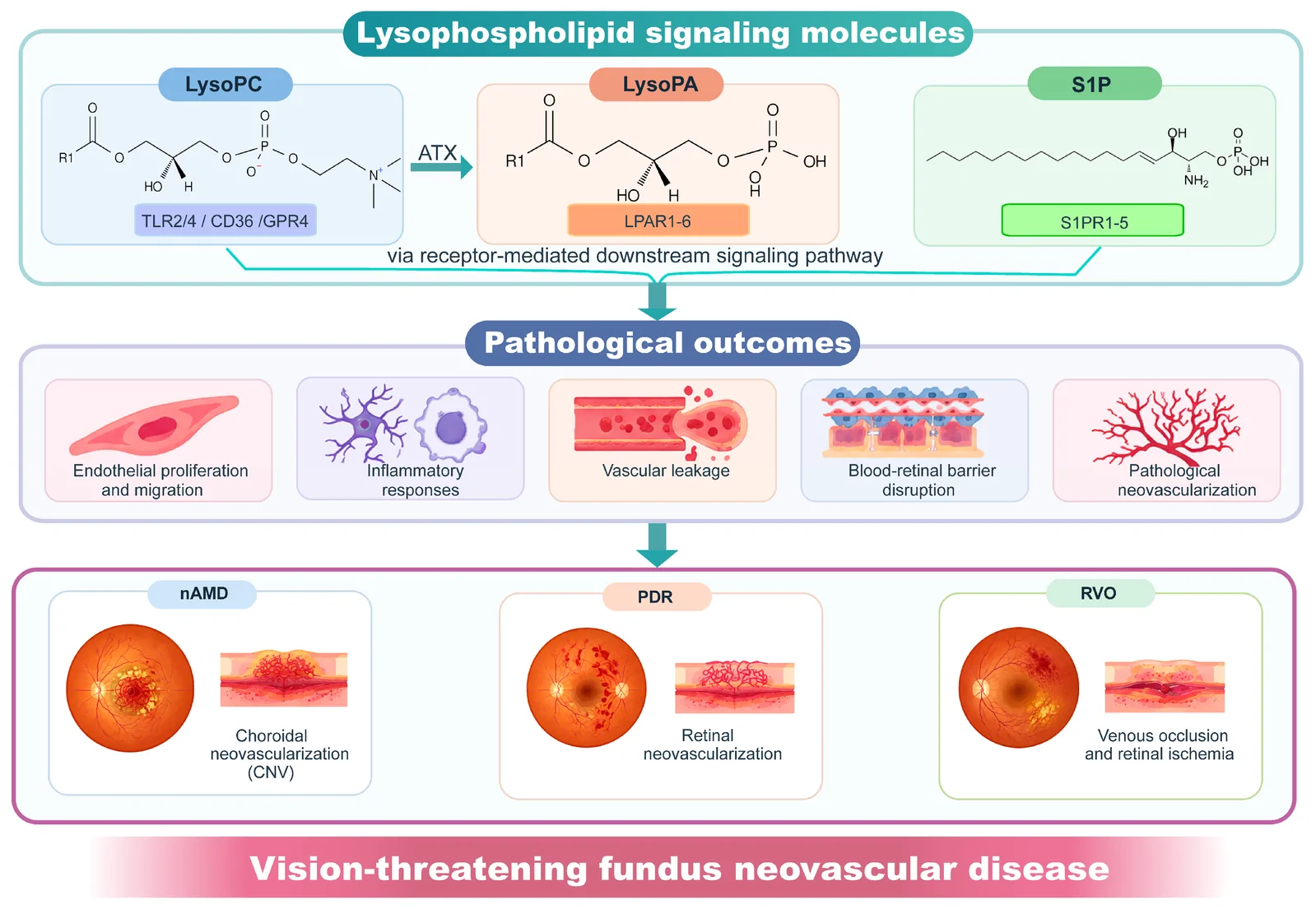
Interconnected lysophospholipid signaling networks contributing to fundus neovascular diseases. LysoPC, LysoPA, and S1P accumulate in the ocular microenvironment and form an interconnected signaling network; LysoPC is converted to LysoPA by autotaxin (ATX). Through distinct receptors and metabolic crosstalk, these mediators promote inflammation, endothelial cell proliferation and migration, vascular leakage, blood–retinal barrier disruption, and pathological neovascularization, driving nAMD, PDR, and RVO. ATX, autotaxin; TLR, Toll-like receptor; CD36, cluster of differentiation 36; GPR4, G protein-coupled receptor 4; LPAR, lysophosphatidic acid receptor; S1PR, sphingosine-1-phosphate receptor; nAMD, neovascular age-related macular degeneration; CNV, choroidal neovascularization; PDR, proliferative diabetic retinopathy; RVO, retinal vein occlusion.

Lysophospholipid signaling molecules, a class of endogenous bioactive lipids generated via the hydrolysis of membrane phospholipids by specific phospholipases, have emerged as increasingly important regulators in pathological angiogenesis. These molecules primarily include lysophosphatidic acid (LysoPA), lysophosphatidylcholine (LysoPC), and sphingosine-1-phosphate (S1P) [10,11], and are released by diverse cell types, including activated platelets, infiltrating inflammatory cells, vascular endothelial cells, and retinal pigment epithelial (RPE) cells. Accumulating evidence indicates that these molecules orchestrate multiple biological processes, including endothelial cell proliferation, migration, survival, and remodeling of the immune microenvironment, thereby exerting profound effects on the maintenance of vascular homeostasis and the modulation of pathological vascular remodeling [10,12]. Through autocrine and paracrine mechanisms, these bioactive lipids selectively engage key membrane receptors, such as G protein-coupled receptors (*GPCRs*) and Toll-like receptors (*TLRs*), thereby activating a broad spectrum of downstream signaling cascades, including *Rho–ROCK*, *PLC–IP3–Ca2+*, *PI3K–Akt*, *MAPK–ERK*, cAMP–protein kinase A (*PKA*), and *Hippo–YAP* pathways. These signaling networks collectively orchestrate pathological ocular angiogenesis and regulate the integrity of the blood–retinal barrier. Therefore, lysophospholipid signaling molecules may represent not only critical pathogenic mediators driving pathological angiogenesis, particularly in retinal neovascular disorders, but also central signaling hubs governing disease initiation and progression [13,14,15,16] (Figure 1).

This review focuses on key lysophospholipid signaling molecules, including LysoPA, LysoPC, and S1P, and systematically summarizes their metabolic pathways, receptor recognition mechanisms, downstream signaling cascades, pro-angiogenic functions, and translational potential as emerging therapeutic targets. By integrating current advances in this field, we aim to provide a theoretical framework and translational rationale for the development of precision intervention strategies targeting lysophospholipid signaling axes.

## 2. Metabolic Pathways of Lysophospholipid Signaling Molecules

### 2.1. Structural and Functional Diversity of Lysophospholipid Signaling Molecules

Based on the nature of their hydrophobic backbone structures, lysophospholipid signaling molecules can be broadly classified into two major categories: lysoglycerophospholipids (LysoGPLs), which are derived from a glycerol backbone and include LysoPA, LysoPC, Lysophosphatidylinositol (LysoPI), Lysophosphatidylethanolamine (LysoPE), and Lysophosphatidylserine (LysoPS); and lysosphingolipids (LysoSLs), which are derived from a sphingosine backbone and primarily comprise S1P and sphingosylphosphorylcholine (SPC).

Although LysoGPLs share a conserved glycerol backbone, they exhibit substantial structural heterogeneity in both their polar head groups and hydrophobic acyl chains [17,18]. The polar head group of LysoPA consists of a phosphate moiety, which undergoes ionization at physiological pH, conferring a net negative charge and acidic properties. In contrast, the choline head group of LysoPC becomes protonated at physiological pH, resulting in a positive charge and alkaline characteristics. At physiological pH, LysoPI, LysoPS, and LysoPE also display distinct and stable ionization profiles: LysoPE exhibits a zwitterionic ionization pattern, whereas LysoPS and LysoPI predominantly exist in an anionic form [19]. These charge-dependent differences contribute to the functional diversification of LysoGPLs, influencing their membrane interaction properties, receptor recognition specificity, and selective activation of downstream signaling pathways. The hydrophobic acyl chains of LysoGPLs further contribute to their remarkable structural diversity, which is primarily determined by variations in acyl chain attachment at the sn-1 or sn-2 positions of the glycerol backbone, carbon chain length (C12–C20), and degree of unsaturation (saturated, monounsaturated, or polyunsaturated). These structural features critically regulate the overall molecular conformation, membrane affinity, and binding affinity for specific target proteins, thereby enabling highly specialized regulation of physiological functions and pathological processes [18,20,21,22].

Unlike LysoGPLs, LysoSLs possess a distinct structural foundation derived from sphingosine, a long-chain amino alcohol. Their core structure consists of a long-chain sphingosine moiety and a phosphate group, giving rise to S1P. Further attachment of a choline group to the phosphate moiety generates SPC. This amphipathic architecture, established through ester-linked molecular interactions, enables LysoSLs to dynamically partition between polar aqueous environments and nonpolar lipid phases, thereby facilitating transmembrane signal transduction and intercellular communication [23,24].

### 2.2. Biosynthetic and Catabolic Pathways of Lysophospholipid Signaling Molecules

**Figure 2:**
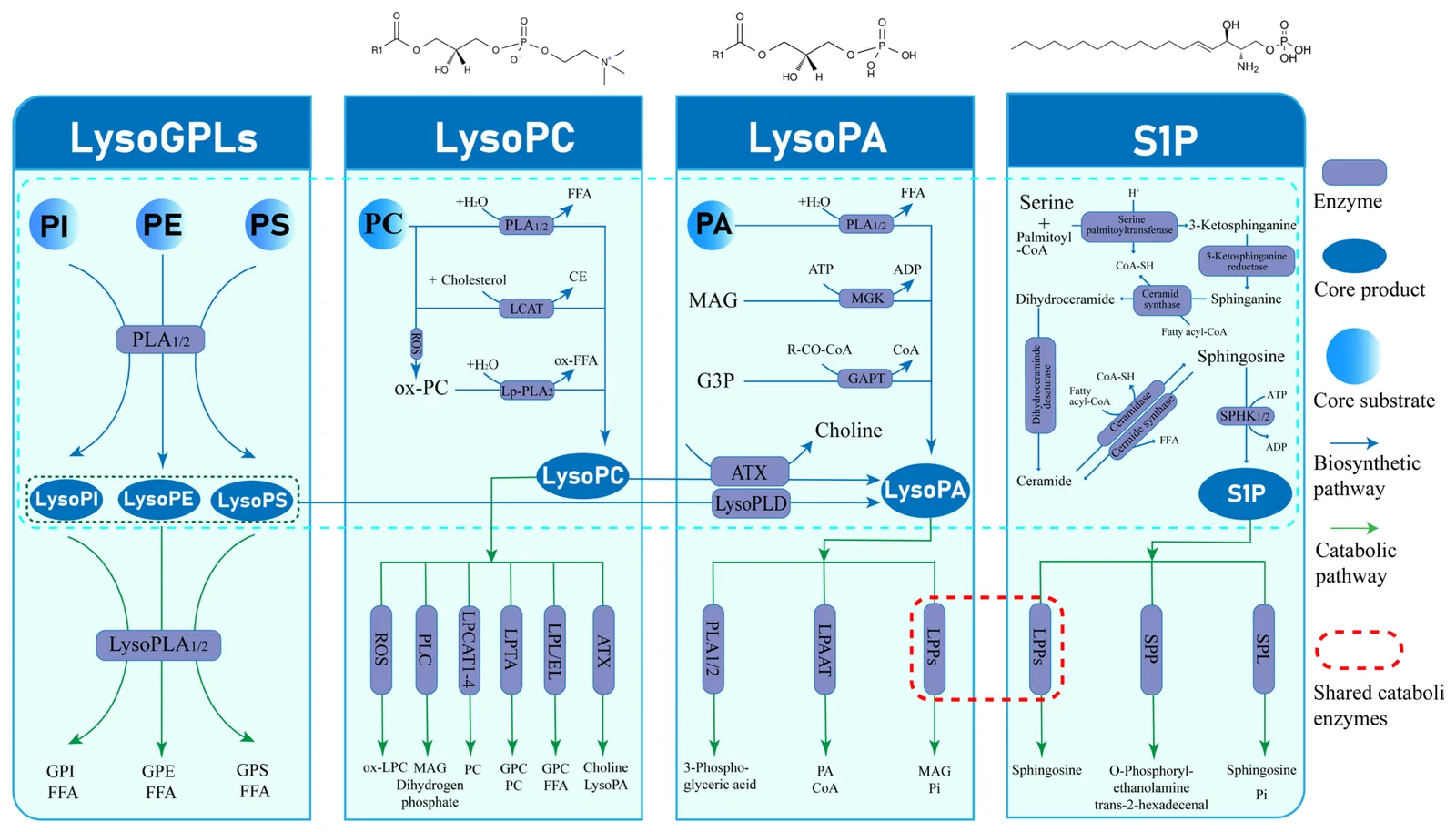
Metabolic pathways of lysophospholipid signaling molecules. lysoglycerophospholipid (LysoGPL)-derived signaling molecules share conserved structural features. Their metabolic pathways also exhibit extensive similarities at both structural and enzymatic levels. These pathways are also functionally interconnected with those of lysosphingolipids (LysoSLs), thereby collectively forming an integrated regulatory network of lysophospholipid signaling governed by common enzymatic mechanisms. Abbreviations: PC, phosphatidylcholine; PE, phosphatidylethanolamine; PS, phosphatidylserine; PI, phosphatidylinositol; PA, phosphatidic acid; MAG, monoacylglycerol; GPC, glycerophosphocholine; GPE, glycerophosphoethanolamine; GPS, glycerophosphoserine; CoA, coenzyme A.

The biological activities of lysophospholipid signaling molecules are largely determined by the dynamic balance between their biosynthesis and catabolism, a balance tightly controlled by key metabolic enzymes (Figure 2). The biosynthesis of LysoPA occurs predominantly through two major routes. (1) Phospholipase D (PLD)-dependent pathway: In this pathway, PLD exhibits high substrate selectivity toward phosphatidylcholine (PC) and catalyzes its conversion into phosphatidic acid (PA), representing the major route of PA generation in mammalian cells [25]. PA is subsequently hydrolyzed by phospholipases A1/A2 (PLA1/2), which remove an acyl chain from the sn-1 or sn-2 position, thereby generating LysoPA [26]. (2) ATX-dependent pathway: This pathway is coupled to LysoPC catabolism, whereby ATX efficiently hydrolyzes circulating LysoPC to produce LysoPA [27]. This reaction represents the predominant extracellular source of LysoPA generation [28,29,30,31]. The catabolism of LysoPA involves several distinct pathways, including: (1) Lipid phosphate phosphatase (LPP)-mediated dephosphorylation: LPPs are a family of integral plasma membrane proteins with extracellularly oriented catalytic domains. They directly hydrolyze extracellular LysoPA, converting it into monoacylglycerol (MAG) through dephosphorylation [27]. (2) Lysophosphatidic acid acyltransferase (LPAAT)-mediated reacylation [32] and (3) soluble epoxide hydrolase (sEH)-mediated hydrolysis [33].

The biosynthesis of LysoPC primarily occurs through the following pathways: (1) PLA1/2-mediated hydrolysis of PC: Based on their biochemical properties, subcellular localization, and activation mechanisms, PLA1/2 can be broadly classified into secreted PLA1/2 (sPLA1/2), cytosolic PLA1/2 (cPLA1/2), calcium-independent PLA2 (iPLA2), and membrane-bound PLA1 (mbPLA1). These enzymes selectively cleave the acyl chains at the sn-1 or sn-2 positions of membrane phosphatidylcholine (PC) molecules, thereby generating LysoPC. In addition, oxidized PC (ox-PC) can be selectively hydrolyzed by specific PLA2 isoforms, such as PLA2G6, resulting in the release of oxidized free fatty acids (ox-FFA) and the generation of LysoPC [34,35]. In oxidized low-density lipoprotein (oxLDL), ox-PC is hydrolyzed by lipoprotein-associated phospholipase A2 (Lp-PLA2), releasing sn-2-specific oxidized free fatty acids (ox-FFA) and generating LysoPC. This process represents a critical molecular link connecting oxidative stress, lipid metabolism, and vascular inflammation [36,37,38,39,40,41,42]. (2) Lecithin–cholesterol acyltransferase (LCAT)-mediated pathway: LCAT specifically catalyzes the transfer of fatty acids from the sn-2 position of PC on the surface of high-density lipoproteins (HDL) to free cholesterol, resulting in the formation of cholesteryl esters and LysoPC [43]. (3) Oxidative stress-driven modification pathway: Within oxidative stress-enriched microenvironments, reactive oxygen species (ROS) attack unsaturated fatty acyl chains within PC molecules, leading to the generation of ox-PC. In addition to PLA2-mediated hydrolysis, oxidatively modified acyl chains of ox-PC can undergo spontaneous non-enzymatic fragmentation, producing short-chain aldehydes and LysoPC [34,35]. The catabolism of LysoPC occurs through several major pathways, including: (1) Lysophospholipase (LPL)-mediated deacylation: LPL hydrolyzes LysoPC to generate glycerophosphocholine (GPC) and free fatty acids (FFA). (2) ATX-dependent pathway: This pathway is coupled to LysoPA biosynthesis and contributes to the metabolic interconversion between lysophospholipid species. (3) Acyltransferase-mediated reacylation: Members of the lysophosphatidylcholine acyltransferase (LPCAT) family utilize acyl-CoA donors to reacylate LysoPC, thereby regenerating PC [34,35].

As important members of the LysoGPL family, LysoPI, LysoPE, and LysoPS share the characteristic monoacyl glycerophospholipid architecture of LysoPC. Accordingly, their metabolic pathways exhibit substantial structural and enzymatic similarities to those of LysoPC. However, differences in their polar head groups confer distinct metabolic fates and biological functions. During biosynthesis, LysoPI, LysoPE, and LysoPS are primarily generated through PLA1/2-mediated hydrolysis of the acyl chains at sn-1 or sn-2 positions of phosphatidylinositol (PI), phosphatidylethanolamine (PE), and phosphatidylserine (PS), respectively, resulting in the removal of one fatty acyl group and the formation of the corresponding lysophospholipids [44,45]. In addition to PLA1/2-mediated metabolism, LysoPI, LysoPE, and LysoPS undergo distinct enzyme-specific metabolic processes. For example, the PLA1 family member DDHD1 exhibits a strong substrate preference for PI species containing polyunsaturated fatty acids (PUFAs) at the sn-2 position, such as arachidonic acid (C20:4) and eicosapentaenoic acid (C20:5). DDHD1 preferentially hydrolyzes the sn-1 acyl chain of these PI molecules, thereby generating PUFA-enriched LysoPI species [46]; moreover, α/β-hydrolase domain-containing protein 16A (ABHD16A) serves as a key biosynthetic enzyme for LysoPS in the brain. Primarily localized to the endoplasmic reticulum, ABHD16A specifically hydrolyzes the sn-1 acyl chain of phosphatidylserine (PS), resulting in the production of LysoPS [47,48]. Regarding their catabolism, LysoPI, LysoPE, and LysoPS can be metabolized through multiple routes. These include lysophospholipase D (LysoPLD)-mediated hydrolysis of the phosphodiester bond to generate LysoPA, as well as lysophospholipase A1/A2 (LysoPLA1/2)-driven deacylation, which produces glycerophosphoinositol (GPI), glycerophosphoethanolamine (GPE), and glycerophosphoserine (GPS), respectively.

S1P biosynthesis represents a critical step in sphingolipid metabolism. The canonical biosynthetic pathway involves the deacylation of ceramide to generate sphingosine, followed by phosphorylation of sphingosine by sphingosine kinases (*SphKs*) to produce S1P. Two major *SphK* isoforms have been identified: *SphK1* and *SphK2* [49,50]. *SphK1* is predominantly localized in the cytoplasm and can translocate to the plasma membrane in response to extracellular stimuli, including growth factors and inflammatory mediators, thereby promoting localized S1P production and secretion. Extracellular S1P subsequently functions as an autocrine or paracrine signaling molecule. In contrast, *SphK2* is primarily localized in the nucleus and endoplasmic reticulum, where S1P generated by *SphK2* mainly regulates intracellular signaling processes. Various cytokines, including tumor necrosis factor-α (TNF-α), interleukin-1β (IL-1β), and *VEGF*, can selectively activate SphKs and consequently promote S1P generation [51]. S1P degradation occurs mainly through two distinct pathways: (1) irreversible cleavage by S1P lyase (SPL), which converts S1P into phosphoethanolamine and trans-2-hexadecenal; and (2) dephosphorylation by S1P phosphatases (SPPs), which regenerates sphingosine and enables sphingolipid recycling [52].

Notably, LPPs function as shared regulatory enzymes within lysophospholipid signaling networks. In addition to catalyzing the dephosphorylation of LysoPA and directly terminating LysoPA-mediated signal transduction, LPPs also dephosphorylate S1P, thereby attenuating S1P-dependent signaling outputs [53]. This enzymatic convergence establishes a functional coupling between the LysoPA and S1P metabolic pathways, creating an integrated lysophospholipid signaling system governed by a common enzymatic framework. Furthermore, LysoPC can be efficiently converted into LysoPA through the “LysoPC-ATX-LysoPA” metabolic axis, whereas LysoPI, LysoPE, and LysoPS can also serve as alternative substrates for LysoPLD-mediated hydrolysis to generate LysoPA [54,55]. These multi-source LysoPA biosynthetic routes not only enhance the abundance and persistence of LysoPA signaling within local microenvironments but also molecularly integrate glycerophospholipid-derived LysoGPLs with sphingolipid-derived LysoSLs, thereby establishing a structurally interconnected and functionally coordinated lysophospholipid signaling network.

### 2.3. Receptors and Signaling Pathways of Lysophospholipid Signaling Molecules

#### 2.3.1. Biological Receptors and Signaling Pathways of LysoPA

Among the LysoGPL family members, LysoPA possesses the simplest chemical structure. Through receptor-mediated signal transduction, LysoPA activates a diverse array of downstream pathways, primarily including four major signaling axes: (1) canonical G protein-coupled receptor (GPCR)-mediated signaling; (2) β-arrestin-dependent signaling; (3) receptor kinase transactivation-dependent signaling; and (4) nuclear receptor peroxisome proliferator-activated receptor-γ (*PPAR-γ*)-mediated signaling. Together, these pathways constitute an intricate and integrated regulatory network. LysoPA receptors (*LPARs*) belong to the classical seven-transmembrane GPCR superfamily. To date, six specific LPAR subtypes have been identified, each exhibiting distinct tissue distribution patterns, signaling properties, and physiological functions [56]. *LPARs* can couple with distinct G protein subtypes, including Gα_i/o_, Gα_q/11_, Gα_12/13_, and Gα_s_, thereby activating multiple downstream signaling networks, such as *Rho–ROCK*, *PLC–IP3–Ca2*^+^, *PI3K–Akt*, *MAPK–ERK*, *cAMP–PKA*, and *Hippo–YAP* pathways. Through these signaling cascades, LPAR activation regulates a broad spectrum of cellular processes, including vascular endothelial cell proliferation and migration, vascular tone regulation, and inflammatory responses [57]. Moreover, individual *LPAR* subtypes exhibit substantial heterogeneity in their ligand-binding affinity and functional activation preferences toward LysoPA. This receptor selectivity is largely influenced by the acyl chain length and saturation status of LysoPA species [58]. Consequently, distinct LysoPA molecular species exert subtype-specific effects on diverse pathophysiological processes, including cell migration, proliferation, survival, morphological remodeling, inflammatory responses, and tissue fibrosis [59,60].

The β-arrestin-dependent pathway represents a non-canonical signaling mechanism that not only mediates *LPAR1* desensitization and internalization but also initiates downstream signaling cascades independently of G protein activation. Among β-arrestin isoforms, β-arrestin 2 plays a predominant role by regulating NF-κB activation and IL-6 expression, thereby directly contributing to chronic vascular inflammation and smooth muscle cell activation [61]. Distinct from the canonical Gα_i_/RhoA/ROCK/NF-κB inflammatory pathway, β-arrestin-mediated signaling promotes G protein-independent NF-κB activation and inflammatory responses, representing an additional mechanism through which LysoPA drives vascular inflammation [62].

Regarding receptor kinase transactivation-dependent signaling, different *LPAR* subtypes exhibit distinct transactivation profiles toward downstream receptor targets. Without requiring de novo ligand synthesis, *LPAR1/3* can transactivate receptor tyrosine kinases, including epidermal growth factor receptor (*EGFR*) and platelet-derived growth factor receptor (*PDGFR*) [63]. In contrast, *LPAR5* can transactivate serine/threonine kinase receptors, such as transforming growth factor-β type I receptor (*TGFBR1*) [64]. These transactivation events amplify signaling programs involved in vascular smooth muscle cell proliferation, migration, and vascular remodeling.

In addition, LysoPA functions as an endogenous ligand for the nuclear transcription factor *PPAR-γ*, directly regulating gene transcription within the nucleus. Notably, LysoPA exhibits context-dependent and bidirectional effects in vascular pathology: on the one hand, it suppresses the expression of inflammatory mediators, including IL-1, IL-6, and TNF-α, thereby alleviating vascular inflammation; on the other hand, it promotes vascular smooth muscle cell proliferation and migration, contributing to vascular wall remodeling and neointimal formation during pathological angiogenesis [65].

Collectively, the coordinated activation of these four signaling modalities enables LysoPA to exert multilayered and multitarget regulation across vascular endothelial cells, smooth muscle cells, immune cells, and platelets, thereby positioning LysoPA as a critical regulator of both physiological and pathological angiogenesis. Beyond GPCR-mediated signaling, LysoPA can also engage non-GPCRs, including transient receptor potential vanilloid 1 (TRPV1) and the receptor for advanced glycation end products (RAGE), indirectly activating downstream pathways such as ROS, NF-κB, and signal transducer and activator of transcription (STAT) signaling. These additional signaling routes further expand the physiological and pathological regulatory repertoire of LysoPA [66].

#### 2.3.2. Biological Receptors and Signaling Pathways of LysoPC

LysoPC is the most abundant lysophospholipid species in plasma and represents a key endogenous bioactive lipid mediator with remarkable context-dependent functions. Depending on the cellular and microenvironmental context, LysoPC can act either as a pro-inflammatory mediator or as an anti-inflammatory and protective factor, highlighting its complex and central regulatory roles in diverse physiological and pathological processes [67]. The biological activities of LysoPC are primarily mediated through the activation or modulation of multiple cell-surface and intracellular receptors, including *GPCRs*, Toll-like receptors (*TLRs*), ion channel receptors, and nuclear receptors. Collectively, these receptors form a complex regulatory network that plays essential roles in a broad spectrum of pathophysiological processes, including lipotoxic inflammation, pathological angiogenesis, cellular proliferation, migration, apoptosis, and immune regulation [68,69,70,71].

Among the receptor systems involved in LysoPC signaling, *GPCRs* serve as the principal mediators responsible for transducing its biological effects. Accumulating evidence has established that LysoPC functions as an endogenous ligand for *GPR4*, activating Gα_q/11_ and Gα_i/o_ proteins to initiate downstream signaling cascades. On one hand, *GPR4* activation promotes intracellular calcium (Ca^2+^) mobilization and protein kinase C (*PKC*) activation, thereby inducing the expression of endothelial adhesion molecules, including intercellular adhesion molecule-1 (ICAM-1) and vascular cell adhesion molecule-1 (VCAM-1), which facilitates monocyte adhesion and vascular inflammation. On the other hand, GPR4 signaling suppresses adenylate cyclase (*AC*) activity and reduces cyclic adenosine monophosphate (cAMP) levels, thereby regulating vascular smooth muscle cell contraction and proliferation and contributing to the initiation and progression of atherosclerosis [57]. LysoPC can also activate other *GPCRs*, including *GPR119*, *GPR55*, and free fatty acid receptor 1 (*FFAR1/GPR40*). Together with *GPR4*, these receptors constitute a more intricate metabolic–inflammatory regulatory network. Through coordinated regulation of key physiological processes, such as intracellular calcium homeostasis, glucose metabolism, and insulin secretion, this network is deeply involved in the pathogenesis and progression of metabolic disorders, further highlighting the pivotal role of LysoPC in orchestrating lipotoxic inflammation and metabolic dysregulation.

As a major phospholipid component of oxLDL, LysoPC plays a central role in orchestrating the crosstalk between innate immunity and lipid metabolism. Studies have demonstrated that, in the absence of classical ligands such as lipopolysaccharide (LPS), LysoPC can directly activate *TLR* signaling pathways, particularly through *TLR4* and the *TLR2/1* heterodimer, leading to NF-κB nuclear translocation and induction of interleukin-8 (IL-8) production. In addition, LysoPC activates the p38 MAPK and c-Jun N-terminal kinase (*JNK*) pathways, but not extracellular signal-regulated kinase (*ERK*), thereby further potentiating its pro-inflammatory effects [3]. However, in the presence of classical ligands such as LPS, LysoPC exhibits pronounced context-dependent and bidirectional regulatory effects. On one hand, LysoPC competitively inhibits the interaction between LPS and *TLR4*, thereby preventing NF-κB nuclear translocation, nitric oxide (NO) production, and inducible nitric oxide synthase (iNOS) expression. Meanwhile, LysoPC suppresses LPS-induced *ERK* phosphorylation, leading to reduced inflammatory mediator release and attenuated inflammatory injury and endotoxemia. On the other hand, LysoPC enhances the expression of LPCAT, which remodels membrane phospholipid composition and promotes *TLR4* translocation into lipid raft domains, thereby increasing the binding efficiency between LPS and *TLR4* and amplifying inflammatory responses. These findings highlight the critical roles of LysoPC and its metabolic enzymes in regulating *TLR4* signaling sensitivity. This dual functionality positions LysoPC as a key regulatory molecule across diverse disease models [72,73,74].

*CD36*, a class B scavenger receptor, is widely expressed in macrophages, endothelial cells, and adipocytes and serves as a critical receptor for the recognition and internalization of LysoPC. It plays a pivotal role in the interplay between lipid metabolism and inflammatory signaling amplification. LysoPC is recognized by *CD36* and internalized into cells through *CD36*-mediated lipid uptake pathways, thereby activating a cascade of downstream protein kinase-dependent signaling pathways, including those mediated by *PKA*, protein kinase B (*PKB*), and *PKC* [75,76,77]. Activated protein kinases subsequently promote the activation of the nuclear transcription factor *PPAR-γ* through phosphorylation. Activated *PPAR-γ*, in turn, transcriptionally upregulates *CD36* expression, establishing a positive feedback loop characterized by “*CD36*-mediated LysoPC internalization → *PPAR-γ* activation → *CD36* upregulation → enhanced LysoPC uptake.” This self-amplifying circuit results in excessive intracellular accumulation of LysoPC, ultimately promoting the secretion of pro-inflammatory cytokines and the generation of ROS, thereby exacerbating oxidative stress and inflammatory injury. Such mechanisms critically contribute to the pathogenesis of lipid toxicity-associated disorders, including atherosclerosis and non-alcoholic fatty liver disease (NAFLD) [78,79].

Furthermore, transient receptor potential (TRP) channels represent a class of lipid-sensitive ion channels that are highly responsive to LysoPC-mediated signaling. LysoPC can modulate TRP channel activity by altering plasma membrane capacitance and fluidity or by directly interacting with the intracellular N-terminal and transmembrane domains of transient receptor potential ankyrin 1 (TRPA1), transient receptor potential vanilloid 2 (TRPV2), and transient receptor potential canonical (TRPC) channels, thereby inducing the formation of functional calcium-permeable channels. This process results in Ca^2+^ influx and subsequent activation of downstream nuclear factor of activated T cells (NFAT) and p38 MAPK signaling pathways, ultimately promoting oxidative stress and inflammatory responses [78].

Collectively, LysoPC functions as an endogenous lipid mediator with dual pro-inflammatory and anti-inflammatory activities. Through coordinated signaling via multiple receptor systems, including *GPCRs*, *TLRs*, *CD36*, and TRP channels, LysoPC establishes a complex regulatory network encompassing calcium signaling, inflammatory pathways, lipid metabolism, and apoptosis. The biological outcomes of LysoPC signaling are determined by the integrated effects of its local concentration, cellular microenvironment, and receptor expression profile. Under physiological conditions, LysoPC contributes to the maintenance of lipid homeostasis, immune equilibrium, and vascular function. However, under pathological conditions, dysregulated LysoPC metabolism or aberrant activation of LysoPC-responsive receptors can drive the progression of inflammation-associated disorders, metabolic diseases, atherosclerosis, and cancer.

#### 2.3.3. Biological Receptors and Signaling Pathways of S1P

Most S1P-mediated biological functions are transduced through a family of specific *GPCRs*, namely S1P receptor1-5 (*S1PR1–5*). Upon ligand binding, these receptors activate heterotrimeric G proteins, predominantly Gα_i/o_, Gα_q/11_, and Gα_12/13_, thereby initiating downstream signaling cascades [13]. Individual *S1PR* subtypes exhibit substantial diversity in their tissue distribution, G protein coupling preferences, and downstream signaling outputs, which underlies their distinct and multifaceted roles in regulating physiological and pathological processes, including cell migration, proliferation, survival, vascular stabilization, and immune cell trafficking [13]. Notably, *LPAR1–3* and *S1PRs* belong to the same endothelial differentiation gene (EDG) subfamily of *GPCRs*. Their ligand-binding pockets may exhibit partial structural overlap, potentially resulting in receptor cross-activation [53,80].

In ocular tissues, *S1PRs* exhibit highly cell-type-specific expression patterns. RPE cells constitutively express *S1PR1–3*, retinal vascular endothelial cells predominantly express *S1PR2/3*, whereas choroidal endothelial cells mainly express *S1PR1* [81,82,83]. Based on existing evidence, *S1PR1* is generally regarded as a key protective receptor in pathological angiogenesis. Through Gα_i/o_-mediated activation of the Akt/eNOS signaling axis and suppression of cAMP production, *S1PR1* enhances endothelial barrier integrity and stabilizes intercellular junctions, thereby restraining aberrant vascular sprouting, inflammatory mediator release, and vascular leakage [84,85]. In contrast, *S1PR2* is considered a major pathogenic receptor. Activation of *S1PR2* through Gα_12/13_ stimulates the *Rho/ROCK–COX-2* pathway, promoting inflammatory responses and disrupting endothelial junctional integrity. Meanwhile, Gα_q_-dependent signaling induces *VEGF* secretion, thereby driving pathological angiogenesis under hypoxic conditions. In addition, *S1PR2* markedly increases the permeability of RPE cells and vascular endothelial cells, contributing to macular edema and hemorrhagic complications. *S1PR3* is preferentially expressed in retinal vascular endothelial cells and is also generally regarded as a pathogenic receptor. It primarily regulates cytoskeletal remodeling through Gα_12/13_-dependent signaling and activates the phospholipase C (*PLC*) pathway via Gα_q_ signaling [86,87,88]. Beyond the canonical plasma membrane receptor-mediated signaling, S1P also exerts receptor-independent intracellular functions. Intracellular S1P can serve as a secondary messenger by directly interacting with specific target proteins. Among these noncanonical pathways, TRAF2 (tumor necrosis factor receptor-associated factor 2)-mediated activation of the NF-κB pathway and amplification of S1P biosynthesis through the PLD–SK1 positive feedback axis represent two particularly important mechanisms, contributing substantially to cell survival, inflammatory amplification, and stress responses [89,90].

**Figure 3:**
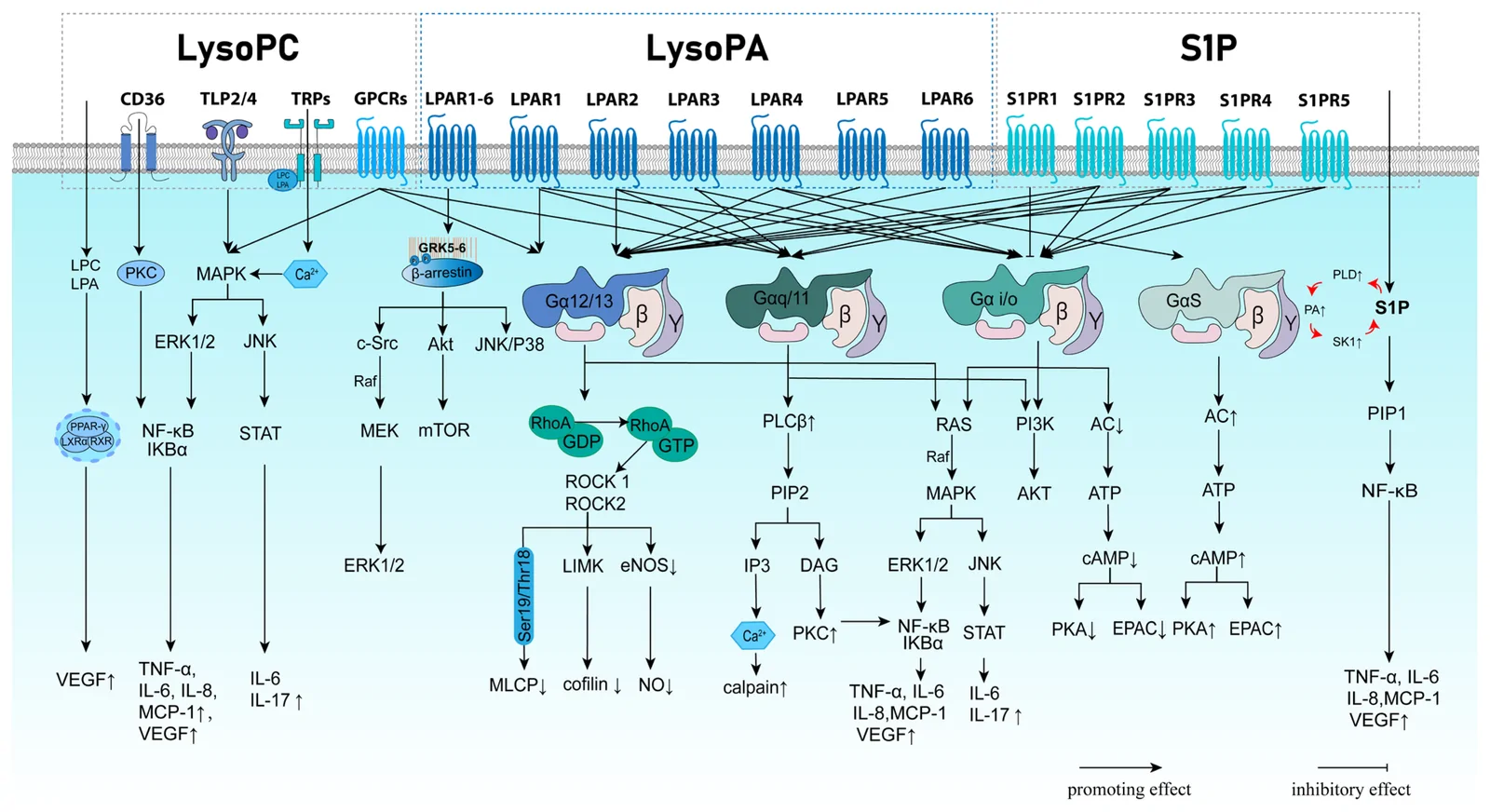
Signaling pathways mediated by LysoPA, LysoPC, and S1P. LysoPA, LysoPC, and S1P exert their regulatory functions through both GPCR-dependent mechanisms and G protein-independent receptor-mediated pathways. Upon activation, S1PRs and LPARs engage their preferential G protein partners to initiate diverse downstream signaling cascades, including the activation of Rho family small GTPases, PLC, PI3K, and AC. These interconnected signaling networks centrally regulate a broad spectrum of pathophysiological processes, including lipotoxic inflammation, pathological angiogenesis, cell proliferation, migration, apoptosis, and immune modulation. AC, adenylate cyclase; PLC, phospholipase C; ROCK, Rho-associated coiled-coil-containing protein kinase; SRF, serum response factor; IP3, inositol 1,4,5-trisphosphate; DAG, diacylglycerol; PKC, protein kinase C; cAMP, cyclic adenosine monophosphate.

Collectively, through an integrated signaling network composed of receptor-dependent and receptor-independent pathways, S1P enables precise regulation of multiple ocular physiological and pathological processes, including angiogenesis, vascular barrier function, and inflammatory responses (Figure 3).

## 3. Pro-Angiogenic Regulatory Effects of Lysophospholipid Signaling Molecules

As an important class of signaling molecules, lysophospholipids exert diverse biological functions through multiple signaling pathways. In FND-associated pathological processes, lysophospholipid-mediated regulation of pathological neovascularization exhibits a remarkable duality, with these molecules capable of both suppressing and promoting aberrant vascular growth. However, under typical pathological microenvironments characterized by persistent chronic inflammation, oxidative stress, and dysregulated lipid metabolism, lysophospholipid signaling predominantly exerts pro-angiogenic effects, thereby driving the initiation and progression of pathological neovascularization.

### 3.1. Changes in the Levels of Lysophospholipid Signaling Molecules in FND Patients

Recent metabolomic and lipidomic studies analyzing vitreous fluid, aqueous humor, and peripheral blood samples from patients with diabetic retinopathy (DR) and nAMD have revealed characteristic dysregulation of lysophospholipid signaling molecules, which is strongly associated with pathological angiogenesis, vascular leakage, and disruption of the local inflammatory microenvironment [91,92]. Among these alterations, LysoPC exhibits a pronounced overall elevation, with LysoPC 16:0, LysoPC 18:0, LysoPC 18:1, and LysoPC 20:4 representing the most significantly altered species. The abundance of these LysoPC molecular species positively correlates with *VEGF* levels, the extent of vascular leakage, and retinal vascular density [93,94]. Moreover, LysoPC elevation is accompanied by coordinated upregulation of *VEGF-A*, *Ang-2*, and LysoPA expression [95,96]. In retinal tissues from patients with reticular pseudodrusen and early-stage DR, substantial accumulation of LysoPC as well as marked increases in S1P levels has been detected, suggesting that bidirectional crosstalk between glycerophospholipid and sphingolipid metabolic pathways plays a critical role in FND pathogenesis [39,97,98,99]. Compared with non-diabetic controls, vitreous samples from patients with PDR exhibit significantly elevated local expression levels of PA, LysoPA, PLA1, *S1PR1*, and sphingosine phosphate lyase 1 (SGPL1). These findings suggest that activation of the PLA1A/AGK signaling axis may enhance PA production and promote abnormal LysoPA accumulation, thereby contributing to the development of pathological neovascularization [100]. Building upon the upstream–downstream relationship between LysoPC and LysoPA within the lipid metabolic network, the LysoPC–ATX–LysoPA–*VEGF* signaling axis has been proposed as a critical driver of pathological angiogenesis. Furthermore, serum levels of LysoPA 18:2 are significantly elevated in patients with polypoidal choroidal vasculopathy (PCV), demonstrating potential diagnostic value in distinguishing affected individuals from healthy controls [101]. In the microenvironment of PDR, the *SphK1/S1P* signaling pathway is aberrantly activated and cooperatively enhances *VEGF* expression, thereby promoting retinal endothelial cell proliferation and migration and exacerbating pathological neovascularization [102].

Collectively, these findings indicate that lysophospholipid signaling molecules tend to accumulate in the ocular microenvironment of patients with FNDs. Through their capacity to regulate multiple signaling cascades, together with the synergistic interactions among LysoPC, LysoPA, and S1P, these lipid mediators exert complex regulatory functions in the central pathological process of ocular angiogenesis. Therefore, targeting lysophospholipid signaling molecules, their metabolic enzymes (such as LPCAT and ATX), or downstream signaling nodes may represent a promising therapeutic strategy for the treatment of neovascular fundus diseases.

### 3.2. Mechanistic Insights into Lysophospholipid Signaling

Studies have demonstrated that LysoPC can be hydrolyzed into LysoPA by ATX, thereby activating *LPAR1–3* receptors and initiating downstream *VEGF*-associated signaling cascades, ultimately establishing the LysoPC–ATX–LysoPA–*VEGF* signaling axis [103]. This signaling axis has been identified by integrated lipidomic and mechanistic studies of ocular diseases as a central regulatory pathway governing retinal neovascular disorders, including PDR and nAMD [16,104,105]. The catalytic activity of ATX is highly context-dependent and jointly regulated by local LysoPC substrate availability, extracellular pH, and hypoxic conditions. Under pathological microenvironments characterized by retinal ischemia or hyperglycemia, retinal endothelial cells and microglia aberrantly upregulate ATX expression, markedly enhancing LysoPA biosynthesis. This process leads to excessive accumulation of intraocular LysoPA and sustained activation of pro-angiogenic signaling pathways.

At the molecular level, LysoPA, the structurally simplest member of the LysoGPL family, exerts multifaceted regulatory effects on retinal endothelial cell behaviors through coordinated signaling mechanisms. Under physiological conditions, LysoPA activates *LPAR4/6* receptors in endothelial cells, triggering the Gα_12/13_–*Rho–ROCK* signaling cascade, which subsequently promotes activation of the YAP/TAZ transcriptional complex. This process suppresses the expression of the Notch ligand Delta-like ligand 4 (DLL4), thereby moderately facilitating endothelial sprouting and vascular network remodeling during embryonic development and normal retinal angiogenesis, while maintaining vascular developmental homeostasis [16]. However, within pathological microenvironments such as PDR and nAMD, the biological functions of LysoPA undergo a disease-associated shift, predominantly exerting pro-angiogenic and pro-inflammatory effects through *LPAR1/2/3*-mediated signaling. On one hand, LysoPA activates the NF-κB inflammatory pathway, inducing the release of pro-inflammatory cytokines including IL-6 and IL-8, while synergistically upregulating *VEGF-A* and *VEGF-C* expression, thereby exacerbating retinal inflammatory infiltration and pathological neovascularization [16]; on the other hand, by modulating the expression of vascular endothelial cadherin (VE-cadherin), LysoPA remodels endothelial junctional architecture and participates in regulating vascular permeability, ultimately contributing to retinal vascular leakage and barrier disruption [16]. Previous cancer-related studies have suggested that LysoPA may exert vascular normalization effects through *LPAR4* signaling. Nevertheless, this mechanism does not appear to represent the predominant response within the retinal pathological microenvironment. Instead, in ocular fundus disorders, LysoPA primarily functions as a promoter of pathological angiogenesis and disease progression [16,106,107]. Furthermore, LysoPA enhances endothelial cell proliferation and survival by activating the *PI3K/Akt* and *MAPK/ERK* signaling pathways while also stimulating the *Rho/ROCK* pathway to reinforce endothelial migration and invasive capacity. Through these multifaceted mechanisms, LysoPA promotes multiple stages of pathological angiogenesis, including vascular sprouting, extension, and remodeling. More importantly, ocular neovascular disorders are characterized by a pathological *VEGF*–ATX–LysoPA positive feedback loop. Elevated *VEGF-A* expression can further induce retinal vascular endothelial cells to upregulate *ATX* and *LPAR1* expression, thereby continuously amplifying LysoPA biosynthesis and downstream signaling activation. This self-reinforcing cascade establishes a vicious cycle that accelerates pathological neovascularization, aggravates vascular leakage, and disrupts the inflammatory microenvironment. Such a mechanism may represent a critical molecular basis underlying the persistent progression and therapeutic refractoriness of retinal neovascular diseases [108].

Microglial activation represents a critical driving mechanism underlying choroidal neovascularization (CNV) in advanced nAMD. Microglia are extensively enriched throughout the course of nAMD lesions and serve not only as a major intraocular source of *VEGF* but also as active regulators of pathological retinal–choroidal vascular remodeling [109]. Animal studies have demonstrated that LysoPC potently induces microglial activation, promoting the transition of microglia from a resting state toward an amoeboid-activated phenotype. This process is accompanied by a marked upregulation of the phagocytic marker *CD68* and selective activation of the NLRP3 inflammasome. Subsequently, through coordinated regulation of the NLRP3/GSDMD/IL-1β pyroptotic axis, together with the canonical inflammatory signaling pathways *ERK1/2* and NF-κB, LysoPC drives programmed pyroptotic cell death in microglia [110]. During pyroptosis, GSDMD-mediated pore formation in the plasma membrane facilitates the extensive release of damage-associated molecular patterns (DAMPs) from the cytoplasm. These signals subsequently recruit and activate peripheral immune cells infiltrating the retina, thereby amplifying local inflammatory responses and ultimately establishing a self-perpetuating vicious cycle of chronic retinal inflammation [111,112,113]. Furthermore, LysoPC functions as an endogenous ligand for *TLR2/4* on monocytes/macrophages [114,115]. Through coordinated activation of downstream mediators, including prostaglandin E2 (PGE2) and adenosine, LysoPC facilitates the polarization of macrophages toward a pro-angiogenic M2d-like phenotype, thereby further exacerbating pathological neovascularization through the paracrine release of angiogenic factors such as *VEGF* [114,116,117]. Meanwhile, as a key lipid metabolic mediator, LysoPC has been hypothesized to participate in the regulation of the ocular inflammatory microenvironment through the gut–retina axis, amplifying local pro-inflammatory and pro-angiogenic responses and synergistically accelerating nAMD progression [118]. In vitro studies have demonstrated that LysoPC exhibits concentration-dependent effects on endothelial angiogenic responses: low concentrations of LysoPC promote endothelial cell proliferation and angiogenesis, whereas excessive LysoPC exposure induces endothelial apoptosis and suppresses angiogenic activity [119,120]. This seemingly contradictory biological duality represents the major reason underlying the ongoing controversy regarding the functional roles of LysoPC. To date, a unified concentration threshold that distinguishes these opposing effects has not been clearly established, and the critical range appears to vary depending on cellular context and LysoPC acyl chain composition. Importantly, metabolomic studies have demonstrated that LysoPC levels are markedly dysregulated in patients with pathological retinal neovascular disorders, including PDR and nAMD. Collectively, these findings suggest that the concentration-dependent bidirectional regulatory mechanism mediated by LysoPC may contribute to the pathogenesis of retinal neovascular diseases while also providing an important theoretical foundation for targeting lipid metabolic pathways as therapeutic strategies against fundus neovascular disorders [91,121,122].

The molecular mechanisms by which S1P regulates pathological angiogenesis are primarily mediated through the coordinated actions of distinct receptor subtypes, including *S1PR1*, *S1PR2*, and *S1PR3*, which collectively contribute to endothelial barrier disruption and aberrant activation of pro-angiogenic signaling pathways. First, under pathological microenvironments characterized by hypoxia, hyperglycemia, and inflammation, *S1PR1* expression is downregulated, resulting in reduced expression of the tight junction protein zonula occludens-1 (ZO-1). Meanwhile, aberrant activation of *S1PR3* similarly downregulates ZO-1 expression, thereby compromising endothelial intercellular tight junction integrity [123,124,125]. In addition, excessive *S1PR3* activation markedly enhances *VEGF-A* expression, ultimately cooperating with other angiogenic signals to drive pathological neovascular lumen formation [124,125]. Second, impairment of ZO-1 function disrupts endothelial barrier integrity, directly increasing vascular permeability. Notably, S1P secreted by pericytes serves as a critical regulator of blood–retinal barrier (BRB) homeostasis. Dysregulation of this signaling axis not only attenuates barrier-protective effects but also triggers the activation of angiogenic mediators, including *VEGF* and transforming growth factor-β (TGF-β), together with their downstream signaling cascades [126]. Furthermore, S1P can suppress the expression of vascular endothelial cadherin (VE-cadherin) through *S1PR2* activation, disrupting the assembly of endothelial adherens junctions. This process further aggravates barrier dysfunction and promotes aberrant endothelial cell proliferation and migration [127].

LysoPC, LysoPA, and S1P represent three central lysophospholipid messengers that mediate extracellular lipid signal transduction. Through metabolic interplay, enzymatic interconversion, and coordinated receptor signaling, these molecules collectively establish a dynamic and highly adaptable lipid signaling network. Among them, LysoPA and S1P exhibit substantial evolutionary and functional homology across multiple dimensions, including molecular structure, metabolic origin, receptor recognition, and biological activity. From a chemical perspective, both molecules are negatively charged lysophospholipids [80]. Metabolically, they are generated through enzymatic remodeling of membrane phospholipids under conditions of cellular stress or membrane restructuring and function as extracellular lipid messengers to mediate intercellular communication. Moreover, both of them share a common degradation pathway mediated by LPPs. At the receptor level, the ligand-binding pockets of their respective G protein-coupled receptors (*LPARs* and *S1PRs*) display a high degree of structural conservation [128]. Cryo-electron microscopy (cryo-EM) analyses have further revealed highly similar ligand recognition conformations between these receptors, providing a structural basis for extensive downstream signaling crosstalk [129]. At the functional level, both lipid mediators can cooperatively activate the Rho–ROCK pathway to regulate cytoskeletal dynamics and engage the PI3K–Akt axis to promote cellular proliferation and resistance to apoptosis [80,130,131]. At the biological level, they exert coordinated effects on diverse cellular processes, including migration, proliferation, survival, morphological remodeling, and intercellular adhesion. At the tissue and systemic levels, they jointly contribute to vascular development, maintenance of barrier integrity, inflammatory responses, and immune cell trafficking [80,130,131]. In contrast, although LysoPC does not directly activate classical EDG family lysophospholipid receptors [80], it participates in signal regulation through two non-canonical mechanisms. First, as a major endogenous substrate of ATX, LysoPC is efficiently converted into LysoPA through the PLD activity of ATX, thereby feeding into the LysoPA signaling axis [132]. Second, LysoPC can selectively engage atypical GPCR pathways, such as GPR119-mediated signaling, to induce indirect signal transduction [133]. Thus, through enzymatic conversion and receptor crosstalk, LysoPC forms a tightly interconnected “precursor-effector” functional module with LysoPA and S1P. This integrated signaling network not only establishes LysoPC-LysoPA coupling and S1P as therapeutic target systems supported by well-defined pathogenic mechanisms but also highlights their potential utility as circulating biomarkers for clinical assessment [80,134,135].

**Figure 4:**
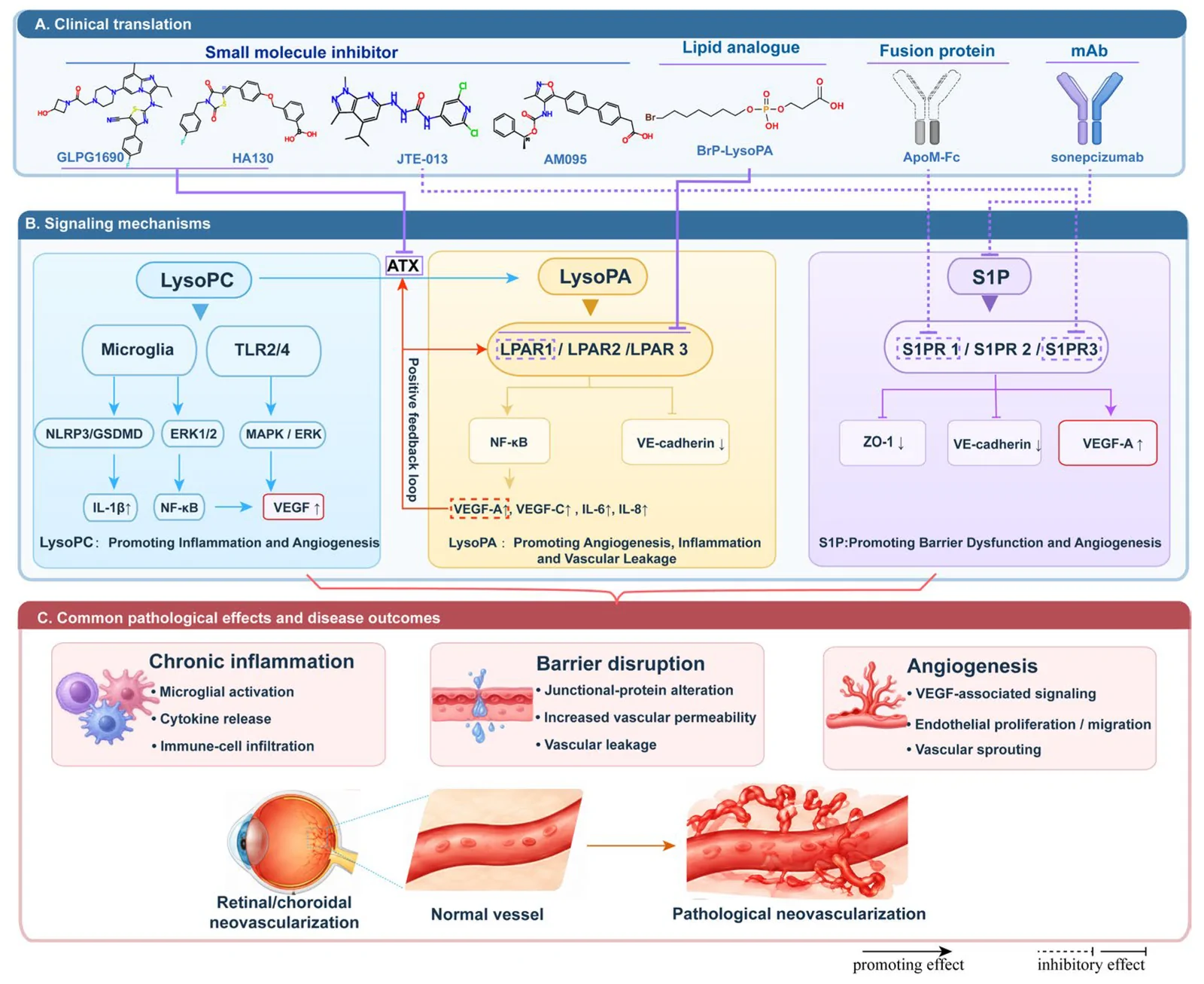
Pro-angiogenic regulatory effects and clinical translation of lysophospholipid signaling molecules. LysoPC, LysoPA, and S1P promote pathological angiogenesis via interconnected signaling networks, collectively driving chronic inflammation, blood–retinal barrier disruption, and pathological neovascularization. These mediators promote immune cell activation, vascular permeability and leakage, and endothelial proliferation and migration, ultimately contributing to retinal and choroidal neovascular diseases. Therapeutic strategies targeting lysophospholipid signaling include small-molecule inhibitors (GLPG1690, HA130, JTE-013, and AM095), a lipid analogue (BrP-LysoPA), a fusion protein (ApoM-Fc), and a monoclonal antibody (sonepcizumab), highlighting the translational potential of targeting these pathways in ocular neovascular diseases. GLPG1690 and HA130, autotaxin inhibitors; JTE-013, sphingosine-1-phosphate receptor 2 antagonist; AM095, lysophosphatidic acid receptor 1 antagonist; BrP-LysoPA, bromophosphonate-LysoPA; ApoM-Fc, apolipoprotein M–Fc fusion protein.

Collectively, LysoPC, LysoPA, and S1P, as three pivotal lysophospholipid signaling mediators, may collectively contribute to the initiation, progression, and recurrence of ocular neovascular disorders, including PDR and nAMD, through intricate metabolic networks and interconnected signaling cascades. This integrated lysophospholipid signaling dysregulation may represent a fundamental pathogenic mechanism underlying pathological vascular remodeling and inflammatory imbalance in retinal diseases (Figure 4B,C).

## 4. Clinical Translational Potential of Lysophospholipid Signaling Molecules as Therapeutic Targets for FNDs

As critical risk-associated mediators in FNDs, lysophospholipid signaling molecules have attracted increasing attention in recent years, with accumulating evidence demonstrating their substantial potential for clinical translation (Figure 4A).

Therapeutic strategies targeting the LysoPC/ATX/LysoPA signaling axis: Among these approaches, the development of ATX inhibitors has emerged as a particularly prominent area of investigation. Structure-guided design of V-type ATX inhibitors (e.g., GLPG1690 derivatives), based on the crystallographic characterization of the ATX catalytic pocket, enables specific coordination with the catalytic zinc ion within the active site, thereby markedly enhancing ATX-binding affinity (with Ki values in the nanomolar range). In vitro studies have demonstrated that these inhibitors effectively suppress ATX-mediated LysoPA production and attenuate downstream *ERK1/2* and Akt phosphorylation. Consequently, they significantly reduce *VEGF* expression and impair LysoPA-induced tube formation and migratory capacity in retinal endothelial cells [136]. Furthermore, the ATX-selective inhibitor HA130 effectively suppresses the aberrant upregulation of ATX expression and enzymatic hyperactivity following cerebral ischemia–reperfusion injury, leading to a marked reduction in the pathologically elevated levels of multiple LysoPA species in both plasma and brain tissues. In parallel, HA130 preserves the expression and spatial distribution of tight junction and adherens junction proteins, thereby significantly attenuating blood–brain barrier (BBB) hyperpermeability, reducing infarct volume, and ultimately maintaining cerebrovascular structural integrity and microenvironmental homeostasis. Given that ATX is not indispensable for adult physiological functions, ATX-selective inhibitors such as HA130 exhibit favorable safety profiles and considerable therapeutic potential. These compounds therefore represent promising candidates for targeting the LysoPC/ATX/LysoPA signaling axis, protecting BBB integrity, maintaining cerebrovascular homeostasis, and preventing ischemic stroke [137]. Importantly, ATX-targeted intervention strategies provide a novel therapeutic avenue to overcome the efficacy limitations associated with current anti-*VEGF* and anti-Ang-2 treatments [138].

Therapeutic strategies targeting the LysoPA–LPAR signaling axis: In the regulation of cerebrovascular function within the central nervous system (CNS), LysoPA-mediated signaling through its receptor *LPAR1* has been established as a key mechanism governing the directional migration of vascular pericytes. This pathway not only provides critical insights into the maintenance of cerebrovascular homeostasis but also offers a cross-tissue conserved molecular basis for understanding retinal microvascular disorders [137,139]. In vitro functional studies using both murine and human primary brain vascular pericytes consistently demonstrate that exogenous LysoPA markedly enhances pericyte migratory capacity [140]. This effect is completely abolished by the selective *LPAR1* antagonist AM095 and the pan-LPAR antagonist BrP-LysoPA [141], indicating that the LysoPA–*LPAR1* axis plays a pivotal role in driving pericyte motility. Aberrant pericyte migration represents not only a major event contributing to blood–brain barrier disruption but also a shared pathological hub linking injury-induced vascular remodeling and fibrotic progression [140,142]. Therefore, pharmacological inhibition of *LPAR1* signaling may represent a promising therapeutic strategy for CNS disorders characterized by BBB impairment, with the potential to stabilize vascular architecture, suppress pathological neovascularization, and limit scar formation [140,142]. As an embryologically homologous extension of the CNS, the retina retains highly conserved mechanisms of LysoPA-dependent vascular remodeling regulation within its microvascular network. Both retinal pericytes and endothelial cells functionally express *LPAR1*, through which LysoPA activates pro-angiogenic and pro-inflammatory signaling programs by engaging surface *LPAR1* receptors on these vascular cells. This signaling cascade disrupts BRB homeostasis, promotes pathological neovascular sprouting, and drives excessive fibrovascular membrane proliferation, collectively representing a critical pathogenic process underlying the development and progression of FNDs, including nAMD and PDR [140,143]. Therefore, pharmacological blockade of the LysoPA–*LPAR1* pathway may simultaneously suppress aberrant retinal pericyte migration, preserve BRB integrity, and restrain pathological neovascular expansion. *LPAR1*-selective antagonists consequently hold considerable translational promise as a novel lipid-targeted therapeutic strategy for FNDs.

Targeting S1P signaling molecules and therapeutic intervention strategies: In recent years, extensive efforts have been devoted to the development of pharmacological approaches targeting S1P signaling molecules and their downstream pathways. Studies have demonstrated that intravitreal administration of the humanized anti-S1P monoclonal antibody sonepcizumab markedly suppresses retinal neovascularization and macrophage infiltration in the oxygen-induced retinopathy (OIR) mouse model, while also reducing the lesion area of laser-induced CNV [15,16,144].

Furthermore, the biological effects mediated by the S1P signaling pathway exhibit pronounced receptor subtype specificity. Among the S1P receptors, *S1PR1* and *S1PR2* often exert opposing effects in the regulation of vascular endothelial function. Endothelial cell-specific activation or overexpression of *S1PR1* markedly suppresses laser-induced CNV formation and attenuates vascular leakage in mice, whereas endothelial cell-specific deletion of S1pr1 gene significantly exacerbates CNV lesion development [81]. Apolipoprotein M (ApoM), the major carrier protein for S1P, not only determines the circulatory half-life of S1P but also facilitates the selective activation of *S1PR1* by S1P [81,145]. Therefore, potentiation of the ApoM–S1P/*S1PR1* axis has emerged as a promising therapeutic strategy. Notably, systemic administration of an engineered ApoM-Fc fusion protein not only achieves CNV suppression comparable to that observed with anti-*VEGF* antibody therapy but also exhibits potent efficacy in limiting pathological vascular leakage [81]. In contrast, activation of S1PR2 is predominantly associated with pro-inflammatory and pro-angiogenic effects. For example, the selective S1PR2 antagonist JTE-013 has been shown to significantly reduce laser-induced CNV lesion size [144]. These findings provide new mechanistic insights and experimental evidence for the development of therapeutic approaches beyond *VEGF* blockade, with the dual capacity to inhibit pathological angiogenesis and restore vascular barrier integrity. Collectively, the S1P–*S1PR* signaling axis represents a highly promising therapeutic target.

In summary, although investigations targeting lysophospholipid signaling molecules in FNDs remain at an early exploratory stage, with substantial evidence derived primarily from in vitro models and studies of systemic diseases such as cancer and fibrosis, their central roles in regulating angiogenesis, inflammatory responses, and cellular migration have been increasingly recognized. As our understanding of the lysophospholipid signaling network continues to advance, this emerging field may provide novel avenues for precision therapeutic intervention in FNDs.

## 5. Conclusions and Future Perspectives

Collectively, lysophospholipid signaling molecules, including LysoPA, LysoPC, and S1P, orchestrate the pathological progression of FNDs through their receptor-specific signaling networks, providing a novel conceptual framework and promising therapeutic targets for disease intervention. Nevertheless, several critical challenges remain unresolved. First, systematic investigations into the spatiotemporal expression dynamics, cell type-specific functions, and signaling network interactions of lysophospholipid molecules in specific FND models are still limited. Second, the effects of these lipid mediators on angiogenesis are highly dependent on the receptor expression landscape within specific tissue microenvironments and on local concentration gradients of bioactive metabolites. Moreover, the complexity of receptor functional specialization remains incompletely understood, and the mechanisms governing receptor homeostasis and dynamic equilibrium during FND progression continue to be largely undefined. Third, cell type-specific mechanisms remain insufficiently characterized. The response profiles of distinct retinal cell populations (e.g., RPE cells, endothelial cells, and microglia) to lysophospholipid signaling have yet to be systematically delineated at single-cell resolution. Fourth, clinical translation remains a major challenge. Currently, comprehensive and well-controlled clinical trials evaluating therapeutic strategies targeting bioactive lipid metabolites in FNDs are lacking, highlighting the urgent need for the development of more precise and effective lipid-targeted interventions. Fifth, current studies mostly treat individual lysophospholipid species as independent signaling molecules, while largely overlooking the functional consequences of structural diversity arising from variations in hydrophobic acyl chains during disease pathogenesis.

Future efforts to overcome these limitations may focus on four major directions. First, the integration of single-cell and spatially resolved lipidomics approaches will enable precise characterization of lipid signaling response profiles and spatial distribution patterns across distinct retinal cell populations. Second, the application of genome-editing technologies to selectively manipulate key lipid metabolic enzymes or receptors will facilitate the elucidation of dynamic mechanisms underlying receptor functional specialization, the establishment of causal relationships between lipid signaling and FND pathogenesis, and the identification of potential therapeutic targets. Third, the establishment of precise relationships between lipid molecular subtypes and their biological functions, together with the development of quantitative structure–activity relationship models, will clarify the metabolic conversion pathways and disease-relevant functions of specific lipid species in ocular tissues. Fourth, innovative therapeutic combination strategies integrating lipid signaling pathway modulation should be developed and evaluated through multicenter, large-scale clinical cohort studies. Comprehensive longitudinal profiling of lysophospholipid signaling molecules and their molecular subtypes in aqueous humor, vitreous fluid, and serum from patients with FNDs may facilitate the establishment of predictive models linking lipid signatures with disease progression and poor responses to anti-*VEGF* therapy, thereby accelerating clinical translation.

LysoPA, LysoPC, and S1P, as key members of the lysophospholipid signaling family, play complex and pivotal roles in the pathological processes underlying FNDs. Although current studies have begun to delineate their pro-angiogenic functions, the sophisticated regulatory mechanisms governing lipid subtype diversity, cell type-specific responses, and receptor functional specialization remain largely unresolved. Future investigations should integrate multi-omics approaches, genome-editing technologies, and next-generation pharmacological strategies to establish a comprehensive evidence framework bridging molecular mechanisms and clinical translation. Precise targeting of lipid metabolic networks may open an entirely new therapeutic dimension for neovascular retinal diseases. A deeper understanding of the fine-tuned regulatory mechanisms within bioactive lipid signaling networks will not only provide critical theoretical insights into the fundamental pathogenesis of FNDs but also facilitate the development of precision intervention strategies based on receptor subtype selectivity and carrier-targeted delivery, thereby offering substantial scientific value and clinical implications.

## Data Availability

No new data were created or analyzed in this study. Data sharing is not applicable to this article.

## Abbreviations

The following abbreviations are used in this manuscript:

FNDs	Fundus neovascular diseases
nAMD	Neovascular age-related macular degeneration
PDR	Proliferative diabetic retinopathy
RVO	Retinal vein occlusion
VEGF	Vascular endothelial growth factor
Angs	Angiopoietins
Ang-2	Angiopoietin-2
RPE	Retinal pigment epithelium
GPCRs	G protein-coupled receptors
TLRs	Toll-like receptors
Rho-ROCK	Rho-Rho-associated coiled-coil-containing protein kinase
PLC-IP3-Ca^2+^	Phospholipase C-inositol 1,4,5-trisphosphate-calcium ion
PI3K-Akt	Phosphatidylinositol 3-kinase-protein kinase B
MAPK-ERK	Mitogen-activated protein kinase-extracellular signal-regulated kinase
cAMP-PKA	Cyclic adenosine monophosphate-protein kinase A
Hippo-YAP	Hippo-yes-associated protein
LysoGPLs	Lysoglycerophospholipids
LysoPA/LPA	Lysophosphatidic acid
LysoPC/LPC	Lysophosphatidylcholine
LysoPI	Lysophosphatidylinositol
LysoPE	Lysophosphatidylethanolamine
LysoPS	Lysophosphatidylserine
LysoSLs	Lysosphingolipids
S1P	Sphingosine-1-phosphate
SPC	Sphingosylphosphorylcholine
PLD	Phospholipase D
PA	Phosphatidic acid
PLA1/2	Phospholipases A1/A2
ATX	Autotaxin
LPP	Lipid phosphate phosphatase
MAG	Monoacylglycerol
LPAAT	Lysophosphatidic acid acyltransferase
sEH	Soluble epoxide hydrolase
PC	Phosphatidylcholine
sPLA1/2	Secreted PLA1/2
cPLA1/2	Cytosolic PLA1/2
iPLA2	Calcium-independent PLA2
mbPLA1	Membrane-bound PLA1
ox-PC	Oxidized PC
ox-FFA	Oxidized free fatty acids
oxLDL	Oxidized low-density lipoprotein
Lp-PLA2	Lipoprotein-associated phospholipase A2
LCAT	Lecithin–cholesterol acyltransferase
HDL	High-density lipoproteins
ROS	Reactive oxygen species
LPL	Lysophospholipase
GPC	Glycerophosphocholine
FFA	Free fatty acids
LPCAT	Lysophosphatidylcholine acyltransferase
PI	Phosphatidylinositol
PE	Phosphatidylethanolamine
PS	Phosphatidylserine
PUFAs	Polyunsaturated fatty acids
ABHD16A	α/β-hydrolase domain-containing protein 16A
LysoPLD	Lysophospholipase D
LysoPLA1/2	Lysophospholipase A1/A2
GPI	Glycerophosphoinositol
GPE	Glycerophosphoethanolamine
GPS	Glycerophosphoserine
SphK	Sphingosine kinase
SPL	S1P lyase
SPP	S1P phosphatases
TNF-α	Tumor necrosis factor-α
IL-1β	Interleukin-1β
LPARs	LysoPA receptors
PPAR-γ	Peroxisome proliferator-activated receptor-γ
NF-κB	Nuclear factor-κB
IL-6	Interleukin-6
EGFR	Epidermal growth factor receptor
PDGFR	Platelet-derived growth factor receptor
TGFBR1	Transforming growth factor-β type I receptor
TGF-β	Transforming growth factor-β
TRPV1	Transient receptor potential vanilloid 1
RAGE	Receptor for advanced glycation end products
STAT	Signal transducer and activator of transcription
PKC	Protein kinase C
ICAM-1	Intercellular adhesion molecule-1
VCAM-1	Vascular cell adhesion molecule-1
AC	Adenylate cyclase
LPS	Lipopolysaccharide
IL-8	Interleukin-8
JNK	c-Jun N-terminal kinase
NO	Nitric oxide
iNOS	Inducible nitric oxide synthase
CD36	Class B scavenger receptor CD36
TRP	Transient receptor potential
TRPA1	Transient receptor potential ankyrin 1
TRPV2	Transient receptor potential vanilloid 2
TRPC	Transient receptor potential canonical
NFAT	Nuclear factor of activated T cells
S1PR1-5	S1P receptor1-5
EDG	Endothelial differentiation gene
ZO-1	Zonula occludens-1
TRAF2	Tumor necrosis factor receptor-associated factor 2
DR	Diabetic retinopathy
PCV	Polypoidal choroidal vasculopathy
SGPL1	Sphingosine phosphate lyase 1
DLL4	Delta-like ligand 4
VE-cadherin	Vascular endothelial cadherin
CNV	Choroidal neovascularization
NLRP3	NOD-like receptor thermal protein domain associated protein 3
GSDMD	Gasdermin D
DAMPs	Damage–associated molecular patterns
PGE2	Prostaglandin E2
BRB	Blood–retina barrier
BBB	Blood–brain barrier
CNS	Central nervous system
OIR	Oxygen–induced retinopathy
ApoM	Apolipoprotein M
